# 889. Early Discontinuations and Adverse Events Among Treatment-Naïve Patients Initiating Integrase Inhibitors in a Real-world Setting

**DOI:** 10.1093/ofid/ofab466.1084

**Published:** 2021-12-04

**Authors:** Charlotte-Paige M Rolle, Jamie Castano, Vu Nguyen, Kiran Patel, Federico Hinestrosa, Edwin DeJesus

**Affiliations:** 1 Orlando Immunology Center, Orlando, FL; 2 Gilead Sciences, Foster City, CA; 3 Orlando Immunology Center, University of Central Florida College of Medicine, Orlando, FL

## Abstract

**Background:**

Cohort studies suggest higher rates of discontinuations (DCs) and adverse events (AEs) with integrase inhibitors (INSTIs) than is reported in clinical trials. Here, we assess DC of different INSTIs in combination with one of two tenofovir prodrugs in the first year following initiation defined as “early DC” in a real-world cohort of treatment-naïve patients.

**Methods:**

This analysis evaluated treatment-naïve patients at a single center initiating raltegravir (RAL), elvitegravir/cobicistat (EVG/c), dolutegravir (DTG) or bictegravir (BIC) in combination with emtricitabine/tenofovir alafenamide (F/TAF) or emtricitabine/tenofovir disoproxil fumarate (F/TDF) between 10/2007-1/2020. Eligible patients had a minimum follow-up of 1 year. The primary endpoint was incidence of early INSTI DC. Secondary endpoints included AEs and risk factors for early INSTI DC and treatment-related AEs.

**Results:**

331 patients were included. Median age was 32 years, 89% were male, 43% were non-White, 8% started RAL-based therapy, 46% started EVG/c-based therapy, 22% started DTG-based therapy and 24% started BIC/F/TAF. 36 discontinued INSTI-based therapy early yielding an incidence rate of 0.17 DCs per person-years (PPY) among RAL patients, 0.14 DCs PPY among EVG/c patients, 0.22 DCs PPY among DTG patients, and 0 DCs PPY among BIC patients, p=0.006. Treatment-related AEs occurred in 27% of RAL patients, 42% of EVG/c patients, 50% of DTG patients, and 43% of BIC patients p=0.607; and were responsible for early DC rates of 0.022 in 3 EVG/c patients and 0.075 in 5 DTG patients. No treatment-related early DCs occurred among RAL or BIC patients. No evaluated factor was significantly associated with early INSTI DC, however DTG use was significantly associated with treatment-related AEs (aOR 3.46, 95% confidence interval: [1.20; 10.82]).

Table 1. Risk factors for early integrase inhibitor discontinuation and treatment-related adverse events

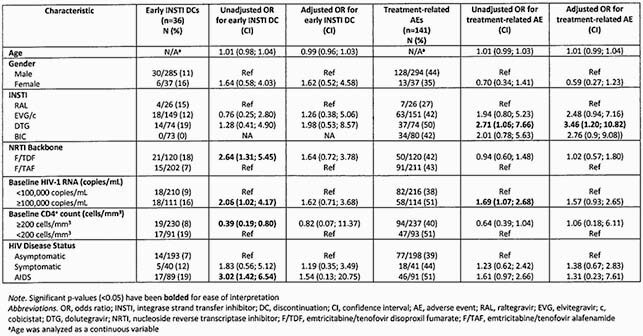

**Conclusion:**

In this cohort, early DCs occurred in 11% initiating INSTI-based therapy, however of these only 2% were treatment-related. These data support use of INSTI-based regimens as preferred options for treatment-naïve patients living with HIV due to their favorable safety and tolerability profiles.

**Disclosures:**

**Charlotte-Paige M. Rolle, MD MPH**, **Gilead Sciences** (Grant/Research Support, Scientific Research Study Investigator, Speaker’s Bureau)**Janssen Infectious Disease** (Scientific Research Study Investigator, Advisor or Review Panel member)**ViiV Healthcare** (Grant/Research Support, Scientific Research Study Investigator, Advisor or Review Panel member, Speaker’s Bureau) **Kiran Patel, PharmD**, **Gilead Sciences** (Employee) **Federico Hinestrosa, MD**, **AbbVie** (Speaker’s Bureau)**Gilead Sciences** (Advisor or Review Panel member, Speaker’s Bureau)**Theratechonologies** (Advisor or Review Panel member)**ViiV Healthcare** (Advisor or Review Panel member, Speaker’s Bureau) **Edwin DeJesus, MD**, **Gilead Sciences** (Scientific Research Study Investigator, Advisor or Review Panel member)

